# Comparative Synthesis of Silver Nanoparticles: Evaluation of Chemical Reduction Procedures, AFM and DLS Size Analysis

**DOI:** 10.3390/ma16155244

**Published:** 2023-07-26

**Authors:** Dan Chicea, Alexandra Nicolae-Maranciuc, Aleksandr S. Doroshkevich, Liana Maria Chicea, Osman Murat Ozkendir

**Affiliations:** 1 Research Center for Complex Physical Systems, Faculty of Sciences, Lucian Blaga University of Sibiu, 550012 Sibiu, Romania; 2 Institute for Interdisciplinary Studies and Research (ISCI), Lucian Blaga University of Sibiu, 550024 Sibiu, Romania; 3 Donetsk Institute for Physics and Engineering Named after O.O. Galkin, NAS of Ukraine, 46, Prospect Nauky, 03028 Kyiv, Ukraine; doroskevich1977@gmail.com; 4 Faculty of Medicine, Lucian Blaga University of Sibiu, 550169 Sibiu, Romania; liana.chicea@ulbsibiu.ro; 5 Faculty of Engineering, Department of Natural and Mathematical Sciences, Tarsus University, Tarsus 33400, Turkey; ozkendir@tarsus.edu.tr

**Keywords:** silver nanoparticles, chemical synthesis, particle sizing, DLS measurements, AFM analysis, localized surface plasmon resonance (LSPR)

## Abstract

The size of silver nanoparticles plays a crucial role in their ultimate application in the medical and industrial fields, as their efficacy is enhanced by decreasing dimensions. This study presents two chemical synthesis procedures for obtaining silver particles and compares the results to a commercially available Ag-based product. The first procedure involves laboratory-based chemical reduction using D-glucose (C_6_H_12_O_6_) and NaOH as reducing agents, while the second approach utilizes trisodium citrate dehydrate (C_6_H_5_Na_3_O_7_·2H_2_O, TSC). The Ag nanoparticle suspensions were examined using FT-IR and UV-VIS spectroscopy, which indicated the formation of Ag particles. The dimensional properties were investigated using Atomic Force Microscopy (AFM) and confirmed by Dynamic Light Scattering (DLS). The results showed particle size from microparticles to nanoparticles, with a particle size of approximately 60 nm observed for the laboratory-based TSC synthesis approach.

## 1. Introduction

In recent years, nanomaterials have evolved to have a variety of biological, electronic, or mechanical applications [1,2]. Nanoparticles have garnered significant attention across diverse fields, including materials science, electronics, medicine, and environmental applications, owing to their exceptional physical and chemical properties at the nanoscale [3]. Their small dimensions and their flexibility led to the development of many approaches for their synthesis, since their size proved to be an important factor in their final purpose [4,5,6,7]. Nanoparticles are complex structures, which can be integrated into innovative simple or layered systems. They can also be used in targeted therapies and possibility integrated into local therapies [8,9], making them relevant to many medical strategies. In biomedical applications, a nanometric size can lead to innovative results by explaining new pathways or new molecule mechanisms. Their size is one of their most important aspects, besides their shape and surface, since the final application depends on it. Their surface is also an essential property, which can determine the trajectories of particles. The chemical functionalization of nanoparticles with more biocompatible layers, for example, is an approach that has recently been studied for advanced medical applications [9,10].

Over the years, metals were often studied for their ability to reflect the light, for their density properties and for their flexibility. Silver nanoparticles (Ag NPs), among other metals, are versatile, possess good electrical and thermal conductivity and have high surface-resonance characteristics [11,12]. In modern science, silver nanoparticles are extremely attractive due to their remarkable antibacterial and inhibitory effects in medical applications [13]. The need for alternative systems for bacteria invasion, in addition to classical antibiotic treatment, in a cost-effective manner, is a global challenge. Silver can more easily be integrated into the new generation of materials than other metallic materials since it has high biocidal activity on pathogens and fungi [14,15]. The mechanism of bacteria destruction can be briefly described in the following steps. The Ag^+^ ions bind to the negatively bacterial membrane, so the lipidic coat is altered. The mitochondria are affected, causing intracellular stress. The bacteria’s reproduction is stopped, and the cytoplasmic content begins to be released while the biofilm is formed [16,17]. Another important aspect of pathogen killing is their high surface-area-to-volume ratio, which allows for them to be such an efficient agent against a broad spectrum of bacteria or viruses [18]. Recently, many studies have been conducted in this regard since bacteria species evolve with high speed, limiting the efficiency of current therapies. Ahmad et al. (2015) [19] synthetized Ag NPs using a phyto approach based on green chemistry. By using Rosa damascena plant as a reducing agent, the researchers showed that biosynthesized spherical Ag NPs have an antibacterial effect on Gram-negative bacteria, even if this effect proved to be smaller than that of penicillin [19]. Green chemistry was also performed by other groups of researchers [20,21,22,23]. Kobylinska et al. (2020) [24] used the hairy roots of two plants to reduce the silver ions and obtain Ag NPs. The study showed that the highest concentration of flavonoids from the leaf plants can lead to the fabrication of spherical, triangular or oval nanoparticles with better antibacterial properties than the initial solution of AgNO_3_ [24]. However, the green chemistry strategy has important shortcomings. Reproductivity is hard to achieve due to the diversity of plants and their growing conditions. Their origin can alter the fabrication process even if the same species is chosen [25]. Another limitation of green chemistry is related to the contaminations that can appear in the sample, since the plants are complex systems that could have adverse effects [26]. Therefore, stable synthesis procedures seem to be preferred for Ag NPs’ fabrication, in which temperature or solvents can be easily controlled.

Ag NPs can also be synthesized via several routes. Chemical or physical approaches allow for them to be obtained in various controllable shapes and sizes [27]. The physical approaches are based on top-down procedures such as laser ablation [28], evaporation–condensation or ion sputtering [29]. Chemical strategies are more frequently studied since they can achieve results in a shorter period. The process of Ag NPs’ fabrication through chemical synthesis is based on the reduction in silver ions from a metal source using reducing and stabilizers agents [30]. Depending on the chosen strategy, Ag NPs can range from a few nanometers up to hundreds of nanometers, even microns. Previous studies showed that the integration of strong reagents in the synthesis, for example sodium borohydride, can assure that low-dimensional nanoparticles with a size range of 8–30 nm are produced, with a high antibacterial effect [31,32,33]. Regardless of the type of procedure that is chosen, one of the most important agents is the surfactant that is introduced, since it is responsible for controlling the size and stability of the final suspension [34]. Chemical reduction offers the advantages of being easily performed at low costs and obtaining high yields in a relatively short time [35,36,37]. Recently, many studies have shown that using certain conditions for the chemical reduction can improve the Ag NPs’ effect in the targeted application [38,39]. Halder et al. (2021) [40] tested the antibacterial effect of TSC-coated and uncoated Ag NPs that were synthetized through chemical reduction and revealed an improvement in the killing rate of coated Ag NPs. The ability of TSC to suppress Ag NPs’ growth has led to a decrease in nanoparticle dimensions of up to 35 nm and to an increased inhibition zone in the case of S. aureus bacteria [40]. In a recent study, Marinescu et al. (2022) [41] investigated the effect that differences in size has on the efficiency of Ag NPs by comparing two different forms of chemical synthesis. The results of the study proved that chemical reductions at room temperature caused the nanoparticles to have a truncated aspect with a maximum size of 20 nm, while in case of solvothermal reaction, the nanoparticles were spherical with an average size of 50 nm and a stronger antibacterial effect. In this study, a higher synthesis temperature was associated with a stronger antibacterial effect on both *E. coli* and *S. aureus* bacteria [41]. Agnihotri et al. (2013) [42] revealed, in a complex experiment, that classical chemical reduction is size- and dose-dependent for *E. coli*, *B. subtilis* and *S. aureus*. The disk-diffusion test performed on *E. coli MTCC 443* strain led to a higher inhibition zone for Ag NPs below 10 nm, with a strong bacteriostatic effect being achieved in a short period of time [42].

The accurate characterization of nanoparticles, particularly their size, is crucial for understanding their behavior and optimizing their performance in fields such as materials science, nanotechnology, and biomedicine. Atomic Force Microscopy (AFM) has emerged as a powerful tool for nanoparticle sizing and characterization, thanks to its exceptional spatial resolution and versatility [43,44].

AFM, a scanning probe microscopy technique, employs a sharp probe that interacts with the sample surface, enabling the acquisition of three-dimensional information at the nanoscale [45]. The advantage of AFM lies in its ability to visualize individual nanoparticles, providing valuable insights into their size, shape, surface morphology, and distribution. Additionally, AFM enables the investigation of dynamic processes such as nanoparticle aggregation, growth, and surface interactions, biological structures and biomolecules, which contribute to a comprehensive understanding of their behavior under different environmental conditions [46,47].

Among the range of characterization techniques that are available, Dynamic Light Scattering (DLS) has emerged as a powerful tool for nanoparticle sizing. DLS is a non-invasive and widely utilized technique that provides valuable insights into the hydrodynamic diameter and size distribution of nanoparticles in suspension [48]. It is based on an analysis of the fluctuations in scattered light intensity arising from the Brownian motion of nanoparticles. By monitoring the temporal intensity fluctuations, DLS enables determination of the particle size distribution, encompassing the average particle diameter and polydispersity index [49].

The accurate sizing of nanoparticles is of the utmost importance, as it directly influences their physical properties, including their surface area, reactivity, and stability, which, in turn, impact their performance in diverse applications [50].

In recent years, DLS has demonstrated great promise for nanoparticle sizing due to its high sensitivity, rapid measurement capabilities, and ability to analyze a wide range of particle sizes [51]. However, challenges arise when applying DLS to nanoparticles, particularly in polydisperse systems, non-spherical particles, and complex suspensions. Factors such as the particle shape, aggregation, and sample preparation approaches can introduce limitations and affect the accuracy of the sizing results [52]. We used a custom-made DLS with time series (TS) data processing procedure that was written by us, and the details are presented in the next section.

The aim of the paper is to study the variance in nanoparticles’ dimensions for further antibacterial applications, and to determine the better choice for their fabrication. In this article, Ag NPs’ preparation was performed using two synthesis approaches. The procedures performed in the laboratory are based on chemical reductions but introduce different reduction agents in the reaction, following a facile chemical synthesis carried out under controllable conditions at room temperature. The study investigates the differences between both procedures and the Ag-77 commercial product, with respect to the Ag NPs’ dimensions, and determines the best way to choose the proper procedure to maintain the dimensions in the nano range, which confers their bactericidal properties [14,53,54,55,56].

## 2. Materials and Procedures

### 2.1. Materials

For this work, the materials used in both chemical syntheses were purchased from Sigma-Aldrich, with only one exception regarding the Acetone ultrapure, which was purchased from Van Waters and Rogers (VWR).

Silver nitrate (AgNO_3_) was chosen with a high purity of ≥99.8% as an ACS Reagent Grade from Sigma-Aldrich, Germany. Alfa-D-Glucose (C_6_H_12_O_6_), anhydrous, with purity of 96%, NaOH with purity of 97%, NaCl with purity ≥99% and trisodium citrate dihydrate (C_6_H_5_Na_3_O_7_·2H_2_O) with 99% purity were all purchased from Sigma-Aldrich, Germany in powder form. The materials were used without further purification.

### 2.2. Chemical Synthesis of Silver Nanoparticles

#### 2.2.1. D-glucose Reduction (AgNP-R1)

**Figure 1:**
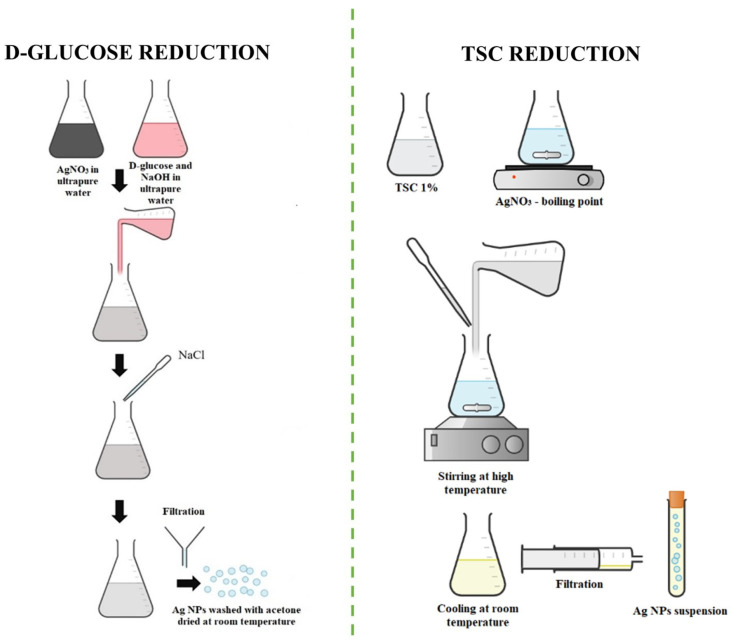
Chemical synthesis of Ag NPs using two different procedures.

The first synthesis was based on the chemical reduction in silver nitrate (AgNO_3_) using D-glucose anhydrous 96% and sodium hydroxide (NaOH), as can be observed in Figure 1. D-glucose reduces the silver ions only in an alkaline medium; therefore, NaOH helps the chemical reduction without interfering with the other chemical reagents. First, 100 mL of 5 mM AgNO_3_ solution was prepared by completely dissolving the silver powder in ultrapure water for 10 min at room temperature. The second step was the preparation of the reduction solution based on D-glucose (C_6_H_12_O_6_) and NaOH. A total of 0.01 M D-glucose and 0.25 M of NaOH were dissolved in 400 mL ultrapure water under magnetic stirring at 82 °C for 30 min. The silver solution was added slowly to the reduction solution until it turned from a colorless color to a light yellow color. After 15 min of mixing, 1.28 M NaCl was added to the solution as a stabilizing agent. The final part of the synthesis was based on the washing steps, realized with ultrapure acetone, before drying at room temperature. The solution was not filtered since, in our attempt to complete this step, the Millex Syringe Pores with 0.22 μm size pores we used quickly clogged. The silver nanoparticle solution was maintained at room temperature for subsequent analysis.

#### 2.2.2. TSC Reduction (AgNP-R2)

The second synthesis procedure that was proposed was also based on chemical reduction, but the reduction agent that was used was tri-Sodium citrate dihydrate (TSC). In the second procedure, TSC was also used as a stabilizing agent in Ag NPs’ formation, so no other chemical reagent was introduced in the reaction. A total of 50 mL of 10 mM AgNO_3_ was heated in an Erlenmeyer glass until the boiling point was reached. In parallel, 1% TSC solution was obtained and added drop by drop to the AgNO_3_ solution by heating and stirring for 10 min. The mixing was stopped when the color changed to yellow, and the Ag NPs solution was left to cool at room temperature. The silver nanoparticle solution was filtered using Millex Syringe Pores of 0.22 μm from Sigma-Aldrich. The final suspension was maintained at room temperature for the next analysis.

#### 2.2.3. Argentum+77 Pure Life Product (Ag-77)

The natural product Argentum+77 from Pure Life company (Pure Life Company, Bucharest, Romania) is a colloidal ionic silver solution with particles distributed in distillated and structured water. The main ingredient of the product is silver 77 ppm (silver purity: 99.99%) mixed with pure water (purity 1 μS/cm). The solution is a homogeneous mix of silver ions with a positive charge (97%) with nanometric particles of colloidal silver in pure water, according to the description of the Agnes Itara, which claims that the product can improve the immune system by eliminating toxins from the skin in medical applications.

### 2.3. Characterization Techniques

Particle size characterization was performed using DLS measurements. AFM was performed on Ag NPs suspension to obtain dimensional information while DLS measurements confirmed the results. FT-IR spectroscopy highlights the proper synthesis conditions for silver nanoparticles by identifying the specific chemical bonds formed in the reactions. The stability of the solution and the formation of Ag NPs were confirmed through UV-VIS spectroscopy.

#### 2.3.1. AFM

AFM is a scanning probe microscopy technique that allows for the high-resolution imaging and mechanical characterization of surfaces at the nanoscale level. The basic principle of AFM is based on the interaction forces between the tip and the sample surface, which include van der Waals forces, electrostatic forces, magnetic forces, and chemical bonding forces, among others. As the tip is brought close to the sample surface, these forces cause a deflection in the cantilever, which is typically measured using a laser beam deflection procedure and measuring the deflection as the difference between the intensity of the beam, recorded by a four-quadrant photo diode. This deflection is then used to control the position of the tip in the vertical direction, maintaining a constant distance between the tip and the sample surface, which is referred to as the “setpoint”.

AFM can be operated in several modes, each with its own advantages and limitations. In contact mode, the tip is scanned across the sample surface while maintaining constant contact, providing high-resolution topographic information [57]. This procedure is straightforward but can leave scars on the sample and wears out the cantilever tip quite quickly, causing a progressive increasing artifact as scanning continues, as will be presented further on in this subsection.

In tapping mode, the tip oscillates near the sample surface at a resonant frequency, providing enhanced sensitivity to surface properties such as stiffness and adhesion [58]. AFM can also be used to measure a variety of the sample’s physical properties, such as its surface potential, magnetic fields, and thermal conductivity [59], among others.

In this work, we used an AGILENT 5500 AFM (Agilent, Santa Clara, CA, USA), with the sample fixed on a substrate and the scanner moving above the sample. A mica substrate was used as substrate. It was cleaved right before sample deposition to assure that the surface was not contaminated by macromolecules or fine dust particles from the air inside the laboratory. Diluted Ag NPs suspension, as described in the subsection presenting the synthesis procedure, was deposited as a small drop and stretched carefully on the mica substrate using a microscope glass coverslip. It was allowed to slowly evaporate, and the Ag NP remained attached to the substrate.

Tapping mode (also named half-contact) was chosen to scan the prepared samples, as described above. The resolution was 512 × 512 pixels. The AFM scanner and sample holder assembly were located on a massive granite thick slab, suspended on the custom Agilent phonically insulated box, to assure isolation from possible vibrations in the building that might occur as people walk through corridors and the possible conversations near the laboratory. Measurements were carried out during the evenings, when few people were in the building. A soft cantilever was chosen with a very sharp tip to reduce scanning artifacts caused by the radius of the tip, which increase as scanning continues and the tip wears out.

The software used to process the acquired AFM topography images was Gwyddion 2.63, running on a Linux Mint platform. The images were processed by subtracting the background, correcting scars, leveling to a three-point plane, and shifting minimum value to zero. Noise filtration was also used by choosing a mean value of five pixels. This filtration improves the quality of the acquired data, because the very soft cantilever tends to oscillate on hard surfaces, creating the appearance of a rough surface for the objects. The noise caused by the amplified oscillation can be reduced by using a stiffer cantilever, which, in turn, creates scars on the topography image and possibly causes small displacements of the deposited objects. With these considerations in mind, we used soft cantilevers with sharp tip for scanning and used filtration to remove the noise.

Another possible source of artifacts is the mica substrate cleaving. After several cleaving operations, it is possible that the atomic layer does not peel uniformly, meaning that more than one layer needs to be peeled on some parts, causing the substrate to have different heights in different portions. This produces artifacts in the scanned image; therefore, NPs located on the edge of such a region were excluded from consideration when assessing the diameter, as further described.

**Figure 2:**
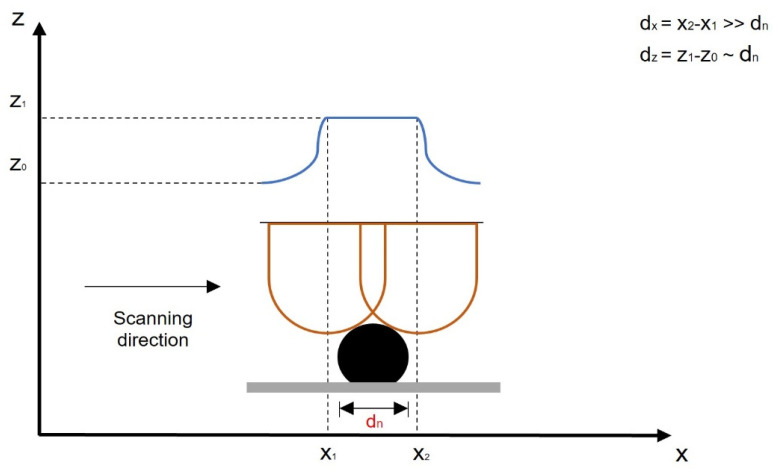
The AFM tip scanning over a NP from left to right and the recorded profile.

When using AFM for more than 3D imaging, as it is used to measure the size of the objects on the substrate, care must be taken to avoid automatic grain size measurements, as shown in Figure 2.

The cantilever tip moves from left to right over the scanning area and the z coordinate (cantilever tip height) is recorded. If we measure the diameter of the grain as the apparent horizontal diameter:

(1) $$d_{horiz}=x_{2}-x_{1}$$

We can easily notice that it is considerably bigger than the actual diameter of the NP, because the tip, no matter how sharp it is in the beginning, wears out during scanning. We then have to scan an NP with a diameter of 20 nm with a tip that has a radius of more than 50–60 nm soon after the preparation, parameter adjustment and scanning begins. To avoid that uncertainty [48], rather than calculating the grain size by using a software that automatically identifies the grain size by horizontal projection on the substrate plane, profiles were manually extracted over grains that were identified on the topography image and saved in a digital form. On each profile, the height of peak was calculated using Gwyddion peak identification and bilateral height measurements, and the height was assigned as the vertical diameter of the NP. This vertical diameter is presented as follows:

(2) $$d_{vert}=z_{1}-z_{0}$$

Figure 2 reveals that *d_vert_* is very close to the actual diameter of the NP, therefore, this was the diameter for each NP on the amplitude (topography) saved file. This procedure was used to assess NP diameter in [47,48,60,61] and revealed a very good consistency with the diameter assessed using an optical technique, which is DLS; therefore, it was also used in the work reported here.

We preferred this manual approach to allowing automatic grain detection, because the result of the grain selection strongly depends on the value that is chosen for a parameter. As Figure 2 illustrates, the average disk diameter, which is the output, is not accurate, but the vertical dimension is much closer to the real grain diameter.

#### 2.3.2. A Brief Overview of the Dynamic Light Scattering (DLS) Data Processing Procedure

DLS TS can be used in a straightforward manner to determine the average diameter of suspended particles [60,62,63,64]. Alternatively, particle size distribution can be estimated using maximum entropy algorithms [65,66] or CONTIN [67,68]. The maximum entropy procedure [65,66] is a fitting-based approach with computationally intensive procedures. CONTIN, which uses the inverse Laplace transform [67,68], requires filtering due to its sensitivity to noise [69] and involves computation-intensive steps [70]. The ill-posed nature of the inverse Laplace transform can lead to ambiguous results unless regularization is introduced, which relies on the choice of an appropriate parameter value. Other techniques involving Artificial Neural Networks (ANN) [71,72] have been used for processing DLS TS, including the averaged scattered light intensity frequency spectrum [73]. ANN models with different inputs, such as autocorrelation [74], have demonstrated faster processing compared to traditional fitting procedures, with improved accuracy. An advancement in the use of ANNs is presented in [75], expanding the particle size range to 6000 nm. This alternative was not used here because it does not offer insight into the mono- or polydispersity of the suspension.

The DLS procedure involves the utilization of a coherent light beam focused on particles suspended in a liquid solvent [76,77,78,79]. The scattered light from the particles is also coherent, resulting in an interference image. A detector measures the interference intensity, and the data acquisition system (DAS) records this information as a time series [76,77]. The processing of the recorded data aims to obtain the average diameter of the suspended particles in a straightforward manner [63,76,77], and is capable of processing a DLS time series obtained from particles in suspension, providing the average size of nanoparticles and microparticles.

**Figure 3:**
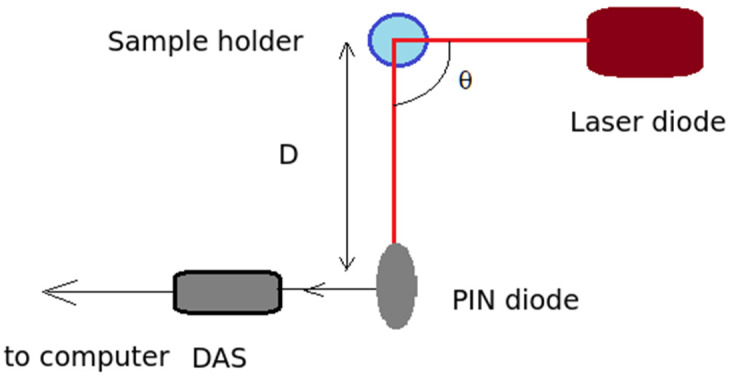
The DLS setup.

Figure 3 illustrates the basic setup of a DLS system. The coherent light source, which, for this work, was a laser diode, emits light at a wavelength of 635 nm. The power source was a rechargeable Pb battery of 7 Ah capacity to avoid any possible electric noise being produced by the modulation of the Laser beam, which might add noise to the recorded time series. The recording angle θ, also called the scattering angle, can be adjusted by changing the detector position from 0 to 180°.

The samples consisted of NPs suspended in solvent. The distance D between the sample and the detector is adjustable to match the average speckle size to the detector size as closely as possible, and was 11 cm for the setup used in the work presented here.

The DLS TS comprises the recorded values obtained by the DAS at a specific sampling rate f, corresponding to time intervals Δt = 1/f. As mentioned in [80], the width of the autocorrelation function of the intensity time series is proportional to the diffusion coefficient, which depends on the diameter of the scattering centers (SC). Previous studies [76,77] and subsequent advancements [48,78,79] have established a relationship between the frequency spectrum and the Probability Density Function (PDF). According to the Khinchin–Kolmogorov theorem [80,81,82], the frequency spectrum of the light intensity (FS), also called the power spectrum (PS), is related to the autocorrelation function of a process. This feature enables an alternative version of processing DLS TS, which begins by computing the Fourier transform of the scattered light intensity, also named the power spectrum density, (PS), using the fast Fourier transform algorithm (FFT) [73].

**Figure 4:**
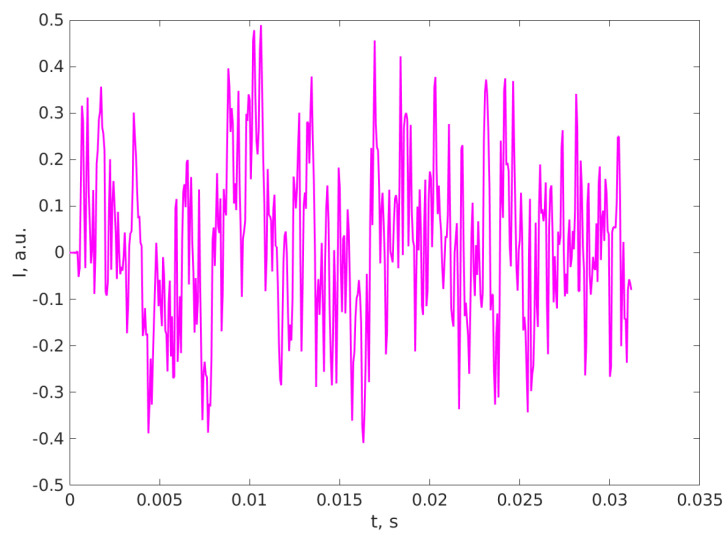
A TS sequence recorded on Ag NPs aqueous suspension. The vertical axis represents I, the recorded signal proportional to the scattered light intensity, expressed in arbitrary units, and the horizontal axis represents the time expressed in seconds.

**Figure 5:**
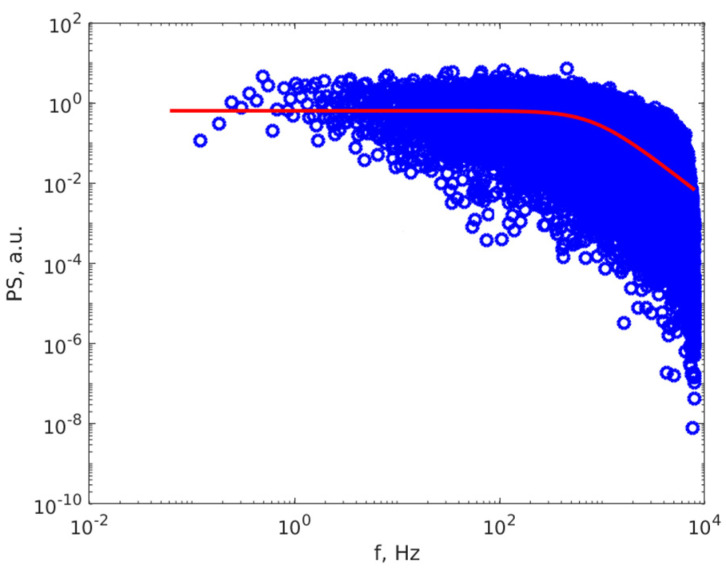
The power spectrum of the time series computed using the FFT algorithm, PS, of the Ag NPs in suspension (blue dots) and the fitted Lorentzian line (red continuous line) versus frequency.

Figure 4 depicts a DLS TS of an Ag NPs suspension and Figure 5 presents the PS calculated using the FFT algorithm.

The PS can be analytically described using the Lorentzian line *S*(*f*) (3) [62,63]:

(3) $$S(f)=a_{0}\frac{a_{1}}{{\left(2\pi f\right)}^{2}+a_{1}^{2}}$$

Equation (3) uses two parameters, *a*_0_ and *a*_1_, which must be determined to best describe the PS computed from the recorded TS using a least squares minimization procedure [62,63]. The average radius of particles in suspension *R* can then be calculated using Equations (4) and (5):

(4) $$R=\frac{2k_{B}Tq^{2}}{6\pi \eta a_{1}}$$

where *q* is the modulus of the scattering vector:

(5) $$q=\frac{4\pi n}{\lambda }\sin\frac{\theta }{2}$$

In Equations (4) and (5), *k_B_* denotes Boltzmann’s constant and *T* denotes the absolute temperature of the fluid.

The DLS sizing procedure continues with a least square fit of the Lorentzian line, described by Equation (3), to the PS calculated using the FFT algorithm. The fit outputs the *a*_0_ and *a*_1_ parameters. The *a*_0_ parameter scales the line to match the plateau on the double logarithmic plot of the PS versus frequency, as illustrated in Figure 5, while the *a*_1_ parameter is used to assess the average diameter of the suspended particles.

Error Calculation for DLS Diameters

As the DLS setup is custom, with all the possible parameters being adjustable to meet particular needs, including the DAS sampling rate, we believe that presenting a brief error analysis is mandatory. To estimate the relative error in assessing the particle radius, *R*, we can substitute Equation (5) into Equation (4) and express the logarithm of *d*, as shown in Equation (6):

(6) $$d=\frac{32\pi k_{B}n^{2}}{3\eta a_{1}\lambda^{2}}T\sin^{2}\frac{\theta }{2}$$

By grouping all the constants together, the differential of that factor becomes zero. Assuming that the sources of errors were the thermodynamic temperature, *T*, and the measuring angle, θ, we can express the logarithm of *d*, as given in Equation (7):

(7) $$\ln\left(d\right)=\ln\frac{32\pi k_{B}n^{2}}{3\eta a_{1}\lambda^{2}}+lnT+2\ln\left(\sin\frac{\theta }{2}\right)$$

By differentiating Equation (7) and considering *dT* and *d*θ as the experimental errors associated with the measurements, assuming errors accumulate in the most unfavorable manner, we obtain Equation (8):

(8) $$\varepsilon_{d}=\frac{\Delta d}{R}=0+\frac{\Delta T}{T}+\frac{1}{\tan\left(\frac{\theta }{2}\right)}\Delta \theta$$

For the temperature measurement, the error was 1 K, with *T* being 20 °C (or 293.15 K). The sampling rate was 16,000 Hz, the detector-tube distance *D* was 11 cm, the scattering angle θ was 90° and the tube diameter was 1 cm, resulting in Δθ, as follows:

(9) $$\Delta \theta =2atan\frac{0.5d_{tube}}{D}$$

Using Equation (9), the calculated relative error was found to be 9.4%. Consequently, the error bars for the diameters were determined using this relative error value.

Another source of error might be the assumption that the particles have a monodisperse size distribution. This assumption is not far from reality, as shown in Figure 5.

Although the error is relatively large, it aligns with the purpose of this study, which aims to utilize a very simple setup and a straightforward data-processing procedure to assess the average diameter of NPs suspended in an aqueous suspension.

#### 2.3.3. UV-VIS

UV-VIS is one of the most-used techniques to characterize Ag NPs. A Specord 210 Plus UV-VIS double-beam spectrophotometer [83] from Analytik Jena company (Analytik Jena GmbH+Co. KG., 07745 Jena, Germany) with a spectral resolution of 2.5 nm was used to read the absorbance between 250 and 700 nm for both synthesis procedures performed in this paper, as well as for Ag-77. The Specord Plus 210 can operate in the range from 185 to 1200 nm with high precision due to the combination of deuterium and halogen lamps, which exhibit a high energy density over the wavelength. All three samples of 1 mL Ag NPs suspensions were introduced in the quartz cuvette, together with a water sample, as a reference. During the recording process, the light beam passes through the Ag NPs suspensions and through the reference sample at the same time, while optical absorbance is collected. The curve in the absorbance obtained in the studied nanoparticles shows the proper yield of the synthesis [84,85]. The use of UV-VIS as a reference for the obtained Ag NPs, starting from silver nitrate, is reported by many other groups in the literature [86,87]. Alvos del Santo et al. (2015) [88] found the absorption for their synthesized Ag NPs to occur at around 420 nm, a specific band for nanoparticles’ formation [88]. Fu et al. (2021) [89] obtained silver nanoparticles in a bright yellow solution using chemical reduction. The yellow color provides the first visual analysis by suggesting that the synthesis process is complete. The results for UV-VIS absorbance revealed a range of 350–400 nm, with a maximum of 400 nm wavelength detection for the synthetized suspension [89].

#### 2.3.4. FT-IR Spectroscopy

The Fourier Transform Infrared spectrophotometer is used in Ag NPs’ structural characterization to determine the functional groups found in the suspension, starting from the reducing agent that was used, and to provide information about the chemical environment of Ag NPs. An ALPHA spectrophotometer from Bruker with an ATR crystal attached was used in this study within a range of 400–4000 cm^−1^ wavelengths at 24 scan times at a resolution of 4 cm^−1^ for both Ag NPs suspensions in transmittance mode. The vibrations of the molecules produced by the infrared irradiation are detected at different wavelengths and shown in the spectra [90]. The spectrum reveals interactions obtained during the synthesis process and shows the adsorption of functional groups on the Ag NPs’ surface. The implementation of FT-IR as characterization procedure for Ag NPs suspensions is reported in other research studies, such as [91,92].

## 3. Results and Discussions

### 3.1. AFM Results

**Figure 6:**
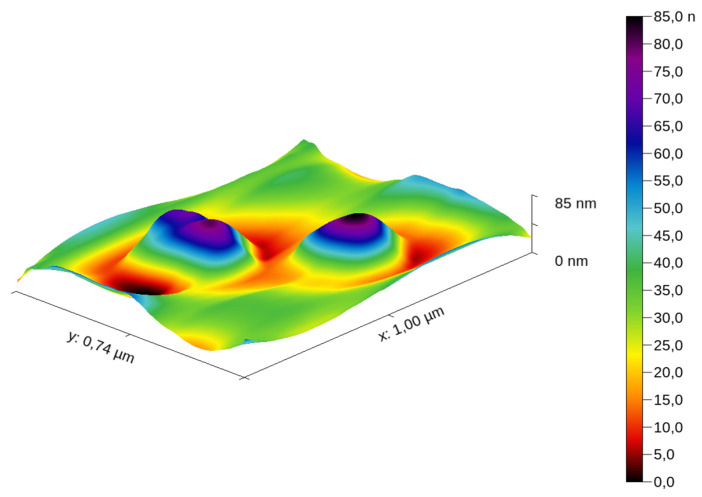
The topography of a region of the sample, illustrating two NPs.

The samples were deposited as described above. Several scans were performed over different regions of the sample prepared, as described in Section 2.3.1, in contact mode and tapping mode. A sample was prepared in the same way but drying was accelerated by placing it on a warm substrate at 50 °C, which resulted in NPs’ agglomeration; therefore, this sample was not used for imaging or extracting profiles. A 3D topography of a scanned region of Ag NPs R2 deposited on the mica substrate is presented in Figure 6.

**Figure 7:**
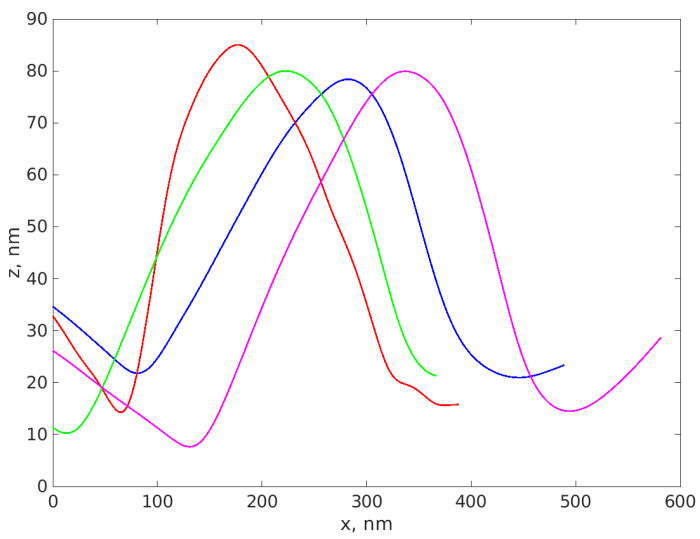
The plot of four profiles extracted over different NPs from the scanned sample, located in different regions of the sample. Z represents the height of the cantilever tip during scanning and x represents the horizontal displacement of the cantilever.

Figure 7 illustrates several profiles extracted over different NPs from the substrate after scanning them.

We notice that the height of the profiles differs, yet remains in the range of tens of nm.

**Figure 8:**
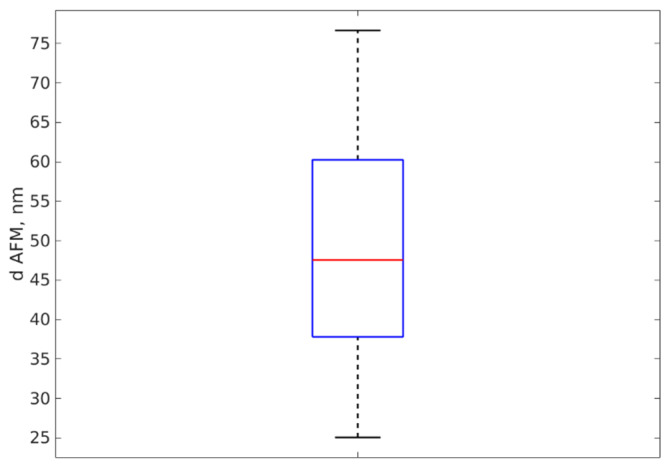
The boxplot of the diameters assessed as the height of the profile peaks extracted over 29 NPs.

A total number of 29 profiles was carefully extracted from the sample and the height of each profile was assessed as described in Section 2.3.1. The average height (and hence the average AFM diameter) was 49 nm and the standard deviation was 14 nm; therefore, we can conclude that the AFM diameter d_AFM_ = 49 +/− 14 nm for AgNP-R2 sample. A boxplot of the diameter values is presented in Figure 8 and illustrates the distribution of the AFM diameter values. The same procedure was used for AgNP-R1 and for Ag-77.

### 3.2. DLS Particle Sizing Results

The described DLS procedure was used to process the TSs recorded for the aqueous suspension of three Ag Np samples, which are AgNP-R1, AgNP-R2 and Ag-77, as described in Section 2. Table 1 describes the average diameter, assessed using the DLS procedure and the AFM procedure.

We should note that the DLS diameters are different from the physical diameter assessed using the AFM procedure. The DLS diameter is actually the diameter of a spherical particle that diffuses at the same rate as the particles that produced the TS. NPs of any shape, including nanorods or even an irregular shape, diffuse in a fluid, and when subject to a coherent light beam, they produce a DLS TS; therefore, the procedure produces a DLS diameter, which should be understood as the hydrodynamic diameter [66,67,68]. Moreover, the hydrodynamic diameter is slightly bigger than the physical diameter for any type of particle, because fluid molecules might be attached to NPs by the electrostatic forces exerted between the surface of the NP, which are typically negative, and the positive part of the polar solvent molecule.

Furthermore, a totally mono-dispersed size distribution is an idealistic concept and cannot be found in the real world, but can be a better or worse approximation of the real particle distribution size. Examining Figure 5, we notice that the fitted Lorentzian line (the red line) describes the PS data very well, which are equally spread around the line, and that the data exhibit a clear plateau with a well-defined turn-over point well, which is an indication that the mono-dispersed approximation is appropriate.

It is important to understand that the average diameter is not the arithmetic average of the diameters, because the intensity of the light scattered by one particle is proportional to the 6th power of the diameter [69,93]. The interference landscape is dominated by the biggest particles in suspension; therefore, if the particles are close to being mono-dispersed, the DLS diameter is actually the average of the hydrodynamic diameter. If the particles are poly-dispersed, the PS is in double logarithmic scale, as in Figure 5, and appears as a sum of Lorentzian lines rather than a single line, with a DLS diameter that is the average of the biggest diameters. This is not the case in our work; the PS is well-described by a single line and the DLS diameter is slightly smaller than the AFM diameter.

The AFM diameter is a confirmation of the size assessed by DLS because the number of profiles is relatively small; if we extract another set of profiles, over other NPs, we obtain a slightly different average diameter, which is still comparable with the presented one. Figure 6 depicts two NPs synthesized using the AgNP-R2 synthesis method.

Considering the significance of the DLS diameter, we can conclude that the two particle-sizing techniques confirm the average diameters we found for the NP synthesized using the two chemical reduction procedures.

### 3.3. UV-VIS Results

**Figure 9:**
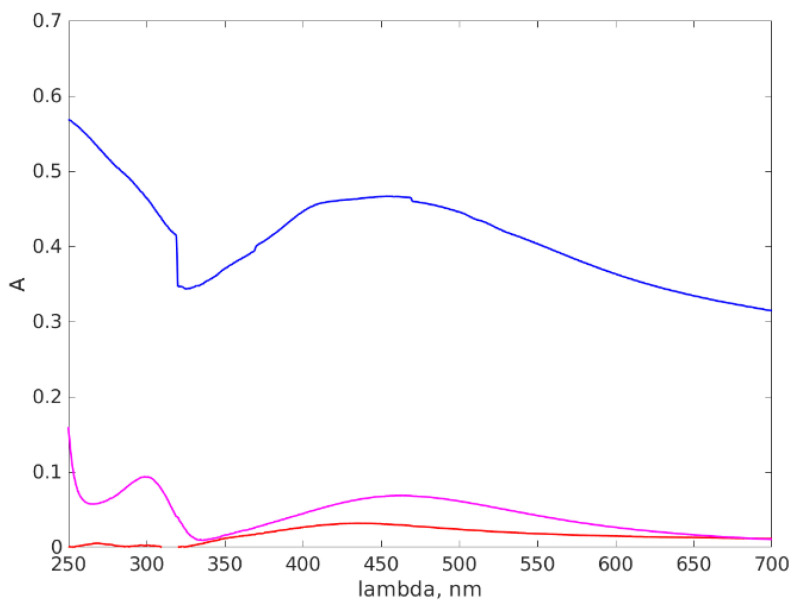
The absorption spectra of the Ag NP aqueous suspensions. The vertical axis represents the absorbance of the suspension and the horizontal axis represents the wavelength. The red line stands for the AgNP-R1 absorption spectrum, the purple line for AgNP-R2 and the blue line for the Ag-77.

Figure 9 illustrates the absorption spectra of the three Ag NPs suspensions we investigated. The UV-VIS absorption spectra of Ag NPs are strongly influenced by their size. As the size of the Ag NPs changes, their localized surface plasmon resonance (LSPR) peak position and intensity in the UV-VIS region can shift and vary. When Ag NPs are excited by incident light, the collective oscillation of conduction electrons in the metal gives rise to LSPR, resulting in the strong absorption and scattering of light at specific wavelengths. The position and intensity of the LSPR peak depends on various factors, including the size and shape of the nanoparticles [94].

In general, as the size of Ag NPs decreases, the LSPR peak shifts towards shorter wavelengths (blue shift). This phenomenon is attributed to the quantum confinement effects, which arise due to the confinement of electrons to a smaller volume. Several studies have reported this blue shift in the UV-VIS absorption spectra of Ag NPs as their size decreases. Reference [95] reports the results of the investigation of the size-dependent optical properties of Ag NPs and observed a systematic blue shift in the absorption spectra as the particle size decreased. Similar observations were made in [94], where they synthesized Ag NPs with varying sizes and observed a consistent blue shift in the absorption spectra with decreasing particle size. There is an intricate interplay between the size, shape, and composition of the nanoparticles, which dictates the shape of the absorption spectra. Additionally, other factors, such as interparticle interactions, surface ligands, and a dielectric environment, can also influence the absorption spectra of Ag NPs [94].

The part of the spectrum for wavelengths smaller than 250 nm was not presented, as it represents the strong absorption of the UV electromagnetic radiation by the water molecules.

Examining Figure 9, we can notice that the Ag-77 absorption spectrum presents a stronger absorption over the whole range, which can be caused by other substances present in solution, which were not disclosed by the producer. All three Ag NPs suspensions present a wide absorption peak at around 450 nm, which is consistent with the position of the absorption peak reported in other references [41,89,94,95,96]. The peaks reported in [41] are located at slightly smaller wavelengths, which is consistent with the smaller size of the Ag NPs that were synthesized. The calculations presented in [94] by several models reveal the influence of the shape in the absorption peak position for 60 nm diameter volume oblate spheroids. The authors state that the bigger the axes ratio, the more shifted towards a smaller wavelength the peak position is. They also present the influence of the polarization direction on the peak position. With these observations in mind, we can conclude that our results are consistent with reports in the literature on similar sizes of Ag NPs and confirm the diameter around 60 nm that we found using AFM and DLS.

Moreover, the absorption peak at 310 nm is caused by the Ag^+^ ions present in solution [41] and we notice that, with AgNP-R2 synthesis, Ag^+^ remained unreduced while, when using AgNP-R1, the ions were reduced.

### 3.4. FT-IR Results

**Figure 10:**
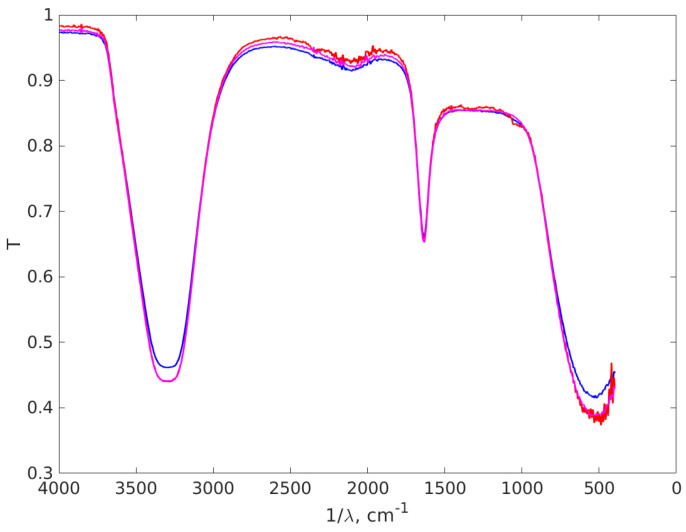
The FT-IR spectra of the Ag NP aqueous suspensions. The vertical axis represents the transmittance of the suspension and the horizontal axis the inverse of the wavelength. The red line stands for the AgNP-R1 absorption spectrum, the purple line for AgNP-R2 and the blue line for the Ag-77.

FT-IR analysis was performed to identify the various functional groups and their implications for Ag NPs’ synthesis, as can be observed in Figure 10. In the case of Ag-77, the FT-IR was performed to compare the results with the procedures performed in the laboratory by our group.

The spectrum of the first sample, AgNP-R1, shows a very large peak at 3310 cm^−1^ associated with the free OH group found in the reduction agent for Ag^+^ to Ag^0^ [97,98]. At 1634 cm^−1^, the sharp peak is assigned to C=O group vibrations, while at 1245 cm^−1^, the C-O- stretching can be found. Both of these effects occur in D-glucose structure and its interaction with Ag^+^ [99,100,101]. The reduction in D-glucose with NaOH is well-known as the Lobry de Bruyn-van Ekenstein rearrangement in chemistry, since the reaction provides a mixture of D-glucose, D-mannose and D-fructose. The presence of OH molecules and C=O groups is also explained by the appearance of these isomers in the reduction reaction [102,103]. For the last peak at 605 cm^−1^, there are several studies in the literature that suggest an association with an Ag network based on Ag–Ag interaction [99,104], but in our opinion this network is quite difficult to obtain and identify through FT-IR analysis, so the peak can be associated with C-H bindings out of the plane, which are found in the molecules [101].

In the spectrum of the second and third sample, AgNP-R2 and Ag-77, the peaks are similar to the ones identified in case of glucose-based synthesis. At 3300 cm^−1^, of O-H stretching vibrations are recorded, since they are found in TSC. The sharp peak observed in 1628 cm^−1^ is correlated with the C=O molecule vibration that appears when the reducing agent acts and forms strong bounds around Ag nanoparticles. The last peak found at 551 cm^−1^ is associated with the C-H bending [101] found at a smaller intensity compared with the first sample due to the high load of this group in the glucose structure.

## 4. Conclusions

In this study, we present two techniques for manufacturing Ag NPs, and the results of the characterization techniques for nanoparticle sizing, focusing on AFM and DLS. We used a nonstandard AFM characterization, and therefore highlighted the details associated with accurate sizing, including the tip wear during scanning and the use of appropriate data-processing procedures. We also described the basic setup of a custom DLS system and explained the processing steps, together with the sources of uncertainty in DLS sizing. Both AFM and DLS revealed that AgNP-R1 produced particles with a wide size distribution, up to one micron. AgNP-R2 produced Ag NPs with a diameter around 60 nm, as shown by the DLS measurements and confirmed by the AFM measurements, consistent with results from the literature for similar reductions. A commercially available aqueous suspension of Ag NPs was investigated, following the same procedure, but both the AFM and the DLS procedures produced Ag particles with a diameter of around 500 nm.

The UV-VIS absorption spectra revealed the existence of the LSPR peak. All three Ag NPs suspensions exhibited a broad absorption peak at around 450 nm, consistent with the literature references. The results align with the findings in the literature for Ag NPs of similar sizes, confirming the diameter of approximately 60 nm determined through AFM and DLS measurements. We also found that AgNP-R2 synthesis Ag^+^ remained unreduced while AgNP-R1 the ions were reduced. FT-IR analysis revealed characteristic peaks associated with functional groups involved in Ag NP synthesis, such as OH groups, C=O group vibrations, and C-O stretching.

Overall, these findings present two simple techniques used for manufacturing Ag particles: one of these is AgNP-R2, for producing Ag NPs with a narrow size distribution of around 60 nm.

## Figures and Tables

**Table 1:** The DLS diameters, the errors that occurred when assessing them and the AFM diameters.

**No.**	**Sample**	**d DLS, nm**	**Δd DLS, nm**	**d AFM, nm**
**1**	**AgNP-R1**	1140	107	973
**2**	**AgNP-R2**	58	6	49
**3**	**Ag-77**	431	41	387

## Data Availability

Tha data we produced by the measurements we made are fully presented in the Table and the Figures presented in the article.
